# Descriptive epidemiology of classical swine fever outbreaks in the period 2013-2018 in Colombia

**DOI:** 10.1371/journal.pone.0234490

**Published:** 2020-06-17

**Authors:** Pilar Pineda, Adriana Deluque, Mario Peña, Olga Lucia Diaz, Alberto Allepuz, Jordi Casal

**Affiliations:** 1 Department Sanitat i Anatomia Animals, Universitat Autònoma de Barcelona, Bellaterra, Barcelona, Catalunya, Spain; 2 Colombian Agriculture and Livestock Institute – ICA, Bogotá, Cundinamarca, Colombia; 3 Centre de Recerca en Sanitat Animal (CReSA), UAB-IRTA, Campus de la Universitat Autònoma de Barcelona, Bellaterra, Barcelona, Catalunya, Spain; Plum Island Animal Disease Center, UNITED STATES

## Abstract

Classical swine fever (CSF) is an infectious viral disease caused by an RNA virus belonging to the Pestivirus genus. A total of 134 outbreaks of CSF have occurred in the last seven years in the North of Colombia. The objective of this study was the characterization of the herds affected by CSF from 2013 to 2018. Most of the outbreaks (95%) occured in backyard piggeries. The principal causes of transmission of CSF were the introduction of infected pigs (38%), movements of people (37%) and unknown origin (13%). The epidemiological relationships with 15 affected farms explained 31 outbreaks. The overall attack and mortality rates were 39% and 32%, respectively. The main clinical signs were high fever (67%), incoordination of movements (54%), and prostration (52%). Seventy-three percent of the herds had not been vaccinated against CSF and 17% had been only partially vaccinated. A spatio-temporal analysis, using a Poisson regression model, revealed two clusters with high risk; the first and largest one from 2014 to 2016 had a relative risk (RR) of 13.4 and included part of the departments of Atlántico, Bolívar, Cesar, La Guajira, Norte de Santander, Magdalena and Sucre; and the second cluster (RR = 9.6 in 2016) included municipalities in the north of the department of Cordoba.

## Introduction

Classical swine fever (CSF) is an infectious viral disease caused by an enveloped RNA virus classified in the family *Flaviviridae*, genus *Pestivirus* [1,2]. It is one of the most important diseases in pigs, with great impact on health and the swine industry [3,4]. Pigs and wild boar are the only natural reservoir. CSF virus (CSFV) can be transmitted both horizontally and vertically. The horizontal transmission occurs through direct contact between infected and susceptible pigs [5]. Additionally, indirect contact by mechanical transmission, by people, equipment, swill feeding, and (illegal) trade of animals and animal products, livestock trucks, slurry, other animals, plays an important role [1]. The contact between feral and domestic pigs is also an important factor for the transmission of the virus. Neighboring premises located within a radius of 500 m of infected farms have a higher risk of infection, and the virus easily spreads on premises located in areas with a high density of pigs [6,7].

In America, CSFV is present in Cuba, Dominican Republic, Ecuador, Haiti, Peru and in certain areas of Brazil and Colombia [8]. Other countries, such as Argentina, Chile or Canada, among others, are recognized as free countries, as well as some areas of Brazil, Colombia and Ecuador [9].

Colombia has a swine census of 5.5 million pigs distributed in 239,199 premises, 88.2% of them are backyard producers that reared 35.8% of the census. CSF was endemic in Colombia until the decade of 2000, when an eradication program reduced the disease to the limits of eradication in 2007. The strains of the outbreaks that occurred between 1998 and 2006 belonged to subgroups 1.1 and 2.2 [10,11].

CSF is a notifiable disease in Colombia, besides the compulsory notification by all producers and professionals of the swine sector, a fundamental component of the passive surveillance is a sentinel surveillance network made up of approximately 5,000 veterinarians and para-veterinarians distributed all over the country. These are specifically trained, and report suspected notifiable diseases. Active surveillance is performed only in free zones or in the process of eradication.

The control program is based on vaccination with a live attenuated-C strain vaccine, control of movements of pigs through a health certificate and checkpoints, and passive and active epidemiological surveillance. The program is developed and managed jointly between the official veterinary service (ICA) and the Colombian Pork Producers Association (Porkcolombia), where the ICA is responsible for the direction and development of measures of epidemiological surveillance, control and eradication of the disease, and Porkcolombia is responsible for the vaccination campaign and provides the vaccine. Vaccination is compulsory in endemic or at-risk areas, and the producers are responsible for the application of the vaccine in the commercial farms, while the backyard piggeries are vaccinated by Porkcolombia workers as a free service [12].

According to ICA contingency plan for CSF, diseased and contact animals in affected premises are sacrificed and disposed of safely, facilities are cleaned, disinfected, and remain without animals for 30 days after disposal of the last sick animal. Partial restocking with sentinel pigs for a 21-day period, is conducted before total repopulation. Protection and surveillance areas of 3 km and 7 km, respectively, around the outbreak are defined, where movements are restricted, and vaccination is carried out depending on the region. Finally, an epidemiological investigation to determine possible epidemiological relationships is undertaken.

Colombia has been divided according to disease status into different areas. Departments recognized as CSF free by the OIE (departments of Antioquia, Caldas, Quindio, Risaralda, Valle del Cauca, and the northern area of Cauca). The self-declared free areas (departments of Amazonas, San Andres de Providencia, Guainia, Guaviare, Vaupes, and Vichada). An area under eradication process without vaccination (departments in the south center of the country). A control zone with vaccination (departments of Atlantic Coast and departments of Arauca, Norte de Santander, Casanare, Nariño and Putumayo that are bordering Venezuela and Ecuador) [13].

The aim of the present study was the description and characterization of the 134 outbreaks of CSF that occurred in Colombia in the period 2013–2018, and the analysis of the temporospatial distribution of the cases, in order to identify the areas of greatest risk.

## Materials and methods

### Country and area under study

Colombia is located in the northwestern region of South America on the equatorial line, with a land area of 1,141,748 km^2^. The country is divided into 32 departments, which are grouped into six naturals regions: Amazon, Andean, Atlantic Coast, Insular, Orinoco, and Pacific.

The outbreaks appeared in the Atlantic Coast region, also known as Caribbean, which has an area of 132.288km^2^ and it is located in the north of the country, bordering with the Caribbean Sea and with Venezuela. Some outbreaks appeared in Norte de Santander and Santander (from the Andean region), very close to the border with Atlantic Coast region and Venezuela.

The Atlantic Coast region, where 126 of the 134 outbreaks occurred, has a porcine census of 1,277,340 pigs (23.2% of Colombian pigs). In this region, there is a high proportion of backyard premises (99.8% of the farms, and 91% of the pig census) (Table 1). These farms are characterized by a small number of animals, low technology, low level of biosecurity, and their unregulated situation. In addition, pigs in marshy areas (swamps of Grande de Santa Marta, Zapatosa, Ciénaga Grande del Sinú, Ayapel, and the Magdalena River) are free ranging during the dry season, and some of them can become feral.

**Table 1:** Census of pigs in the CSF affected departments of Colombia.

Department	Number of commercial farms—2018	Census pigs in commercial farms—2018	Number of backyard premises—2018	Census pigs in backyard premises—2018	Proportion of backyard premises	Proportion of backyard pigs
Atlántico	125	85.725	5.213	87.328	97.7%	50.5%
Bolivar	6	5.556	14.505	123.614	100.0%	95.7%
Cesar	29	4.405	7.844	72.973	99.6%	94.3%
Córdoba	18	10.770	41.379	369.440	100.0%	97.2%
La Guajira	-	-	2.617	42.287	100.0%	100.0%
Magdalena	17	5.668	15.040	255.100	99.9%	97.8%
Sucre	64	6.493	28.810	207.981	99.8%	97.0%
**Total Atlantic Coast**	**259**	**118.617**	**115.408**	**1.158.723**	**99.8%**	**90.7%**
Norte Santander	8.676	65.228	4.961	19.170	36.4%	22.7%
Santander	41	13.364	3.129	63.185	98.7%	82.5%
**Total Others**	**8.717**	**78.592**	**8.090**	**82.355**	**48.1%**	**51.2%**
**Grand Total**	**8.976**	**197.209**	**123.498**	**1.241.078**	**93.2%**	**86.3%**

Source: Census of pigs—National Livestock Census– 2018—Epidemiological surveillance -ICA [14]

The departments of Norte de Santander and Santander have a census population of 160,947 pigs, of which 51.2% are backyard pigs. These departments are characterized for having more commercial premises with a medium biosecurity level.

### Data gathering

All confirmed CSF outbreaks declared in the country between 2013 and 2018 were included in this study. The information of each outbreak was obtained by veterinarians of the official veterinary services (ICA) using two questionnaires: the first one (included in the Suplementary Material) was completed on the notification of the suspicion of the outbreak, and the other one during the follow-up of the outbreak. These questionnaires included the disease notification date, characteristics of the farm, vaccination, animals affected by groups, clinical signs and movements of animals and people. The veterinary officer that completed the questionnaire also asked about routes of disease introduction and epidemiological links between premises.

We performed a descriptive analysis of the different variables associated with the morbidity and mortality rates, clinical signs, the transmission mechanisms of the virus, and response to the outbreak. The most probable routes of CSF introduction and epidemiological links between premises were determined by taking into account the information in the questionnaires and records of swine movements. For the determination of the causes of introduction, in outbreaks where two or three causes were possible, the contribution of each one was divided by the number of causes, i.e. if the cause could be either animal movements or neighbors, both causes were scored 0.5. In the same way, in the case of three possible causes each of them was scored 0.3. If more than 3 causes were possible the most likely cause of infection was considered as unknown.

When the infection affected different backyard premises that shared environment and management practices, the small village was considered an epidemiological unit, and defined as a single outbreak.

#### Ethics statement

This study did not need any ethical approval, as it did not include samples or experiments on people. It only included data collected by Department of Animal Health veterinary officers during the epidemiological survey in the outbreaks. Data about identification of the premises and localization further than the municipality were not analyzed in order to avoid the association of any data with the premise where it was obtained.

### Spatio-temporal analysis

A Poisson regression model (SaTScan version 9.6 program) was used to identify possible temporospatial clusters. The model takes into account the number of CSF outbreaks in relation to the number of pig premises by year at risk. The 134 outbreaks that occurred in the period between 1 January 2013 and 31 December 2018 were analyzed. The data were obtained from ICA [14,15]. Only clusters with statistical significance (p <0.05) were reported, due to the excess of cases observed over the expected ones. The cartographic representations were made using the QGIS 3.4 program.

## Results

### Affected departments and premises

Between 2013 and 2018, 134 outbreaks of CSF affected the Northern part of Colombia, with almost half of them (63; 47%) occurring in 2015 (Table 2 and Fig 2). The most affected departments were Magdalena and Cesar. All the outbreaks occurred in departments of the Atlantic Coast region, except the 5 and 3 cases that occurred in Norte de Santander and Santander, respectively, belonging to the Andean region.

**Table 2:** Distribution of CSF outbreaks in Colombia.

Departments	2013	2014	2015	2016	2017	2018	TOTAL
Atlántico	-	-	3	4	5	-	12 (8.9%)
Bolívar	-	2	14	4	-	1	21 (15.6%)
Cesar	8	5	12	1	1	-	27 (20.1%)
Córdoba	-	-	-	11	-	1	12 (8.9%)
La Guajira	1	-	1	-	-	-	2 (1.5%)
Magdalena	-	15	20	5	1	-	41 (30.5%)
Norte de Santander	-	2	-	3	-	-	5 (3.7%)
Santander	-	-	3	-	-	-	3 (2.2%)
Sucre	-	-	10	-	1	-	11 (8.2%)
**Grand Total**	**9 (6.7%)**	**24 (17.9%)**	**63 (47%)**	**28 (20.9%)**	**8 (5.9%)**	**2 (1.5%)**	**134**

The affected departments are characterized by a high proportion of backyard units (93.2%), which are consequently the most affected (127 outbreaks, 94.8% of the pig units). One of the seven farms classified as commercial that became infected had 460 sows, the other were small premises with 4–34 sows or less than 22 fatteners.

Seventy two percent of the backyard’s piggeries (91 premises) were located in urban and semi-rural areas, while 28% (35) were located less than 1 km far from bodies of water (swamps of the Colombian Atlantic Coast or Magdalena River).

### Detection of CSF outbreaks and response times

Most of the outbreaks (84%) were detected by passive surveillance: 69 cases (51%) were notified to the Health Service by the farmers, 23 (17%) by the voluntary sentinel networks and 21 (16%) by third parties. Another 21 premises (16%) were detected by active surveillance due to epidemiological relationships with the outbreaks. The 113 outbreaks detected by passive surveillance were the result of 945 suspicions of CSF declared in the affected regions between 2013 and 2018.

The time elapsed between the identification of clinical signs by the producer and the official declaration of the outbreak was long, especially in the first years. During the 6 years, the median of this period was 29 days (minimum 7; maximum 92); and in the last two years (2017–2018) it was reduced to 15 days (minimum 7; maximum 38).

The two critical points during the 6 years were, the time between the identification of clinical signs by the producer or sentinel and the notification to the official veterinary service (median of 11 days, range 0 to 72), and the period between the official veterinary inspection and the confirmation by the National Veterinary Diagnostic Laboratory (median of 15 days, with a range of 4–78). In the two last years it was reduced to 5 (1 to 28) and 7 days (4 to19), respectively. The period between the notification and official veterinary inspection was 1 day (range 0 to 4).

### Routes of transmission of CSFV and epidemiological links

The most frequently cited routes of transmission of CSFV between the outbreaks were the introduction of infected pigs (cited in 83 outbreaks) and the movement of people (cited in 77 cases). In 17 premises (13%) the origin was unknown (Table 3). Other causes, such as grazing with feral pigs or livestock vehicles were also cited but only in cases where more than one cause was considered.

**Table 3:** Possible causes of the introduction of the CSFV into the epidemiological units according to questionnaires and records.

Transmission route	Number of possible causes indicated in the questionnaire			Total /Weighted percentage*
	1	2	3	
Introducing pigs	23	52	8	(52) 39%
People	24	46	7	(49) 37%
Unknown	17			(17) 13%
Grazing (swamps)		17	8	(11) 8%
Livestock trucks		4	2	(2.7) 2%
Domestic animals	1			(1) 0,7%
Neighborhood herds			2	(0.7) 0.5%
Swill feeding		1		(0.5) 0.4%

* The percentage was calculated attributing a weight of 1, 0,5 and 0,33 if 1, 2 or 3 causes were indicated respectively. e.g. for introducing pigs: (23 x 1 + 52 x 0.5 + 8 x 0.33) = 52

The index case appeared in the municipality of Urumita (Cesar) in June 2013, near the border with Venezuela. According to the epidemiological data, the most probable origin of this outbreak was an illegal introduction of infected pigs from Venezuela.

Epidemiological links were established only in 31 outbreaks (23%), which can be related to 15 affected premises that could be the origin of the infection. All these relationships were between premises of the same or neighbor municipalities.

### Attack and mortality rates and clinical signs

The overall attack and mortality rates were 39% and 32%, respectively (Table 4). The highest proportion of sick pigs (48%) occurred in animals aged 2–6 months, with sows having a lower attack rate (21%). The differences between groups were significant (p <0.001), except when comparing piglets younger than 6 months and adult males. The case-fatality rate in the whole population was 83%, with no differences between age groups.

**Table 4:** Morbidity and mortality rates due to CSF in the 134 outbreaks, classified by age and gender.

Category	# Animals	Diseased	Deaths	Vaccinated	% Attack rate	% Mortality	% Case-fatality
Weaning Pigs <2 months old	3.054	1.174	990	146	38	32	84
Fatteners, 2–6 months old	2.721	1.300	1.076	404	48	40	83
Males >6 Months old	350	138	110	45	39	31	80
Sow >6 Months old	1.296	273	225	583	21	17	82
**Total**	**7.421**	**2.885**	**2.401**	**1.178**	**39**	**32**	**83**

**Fig 1:**
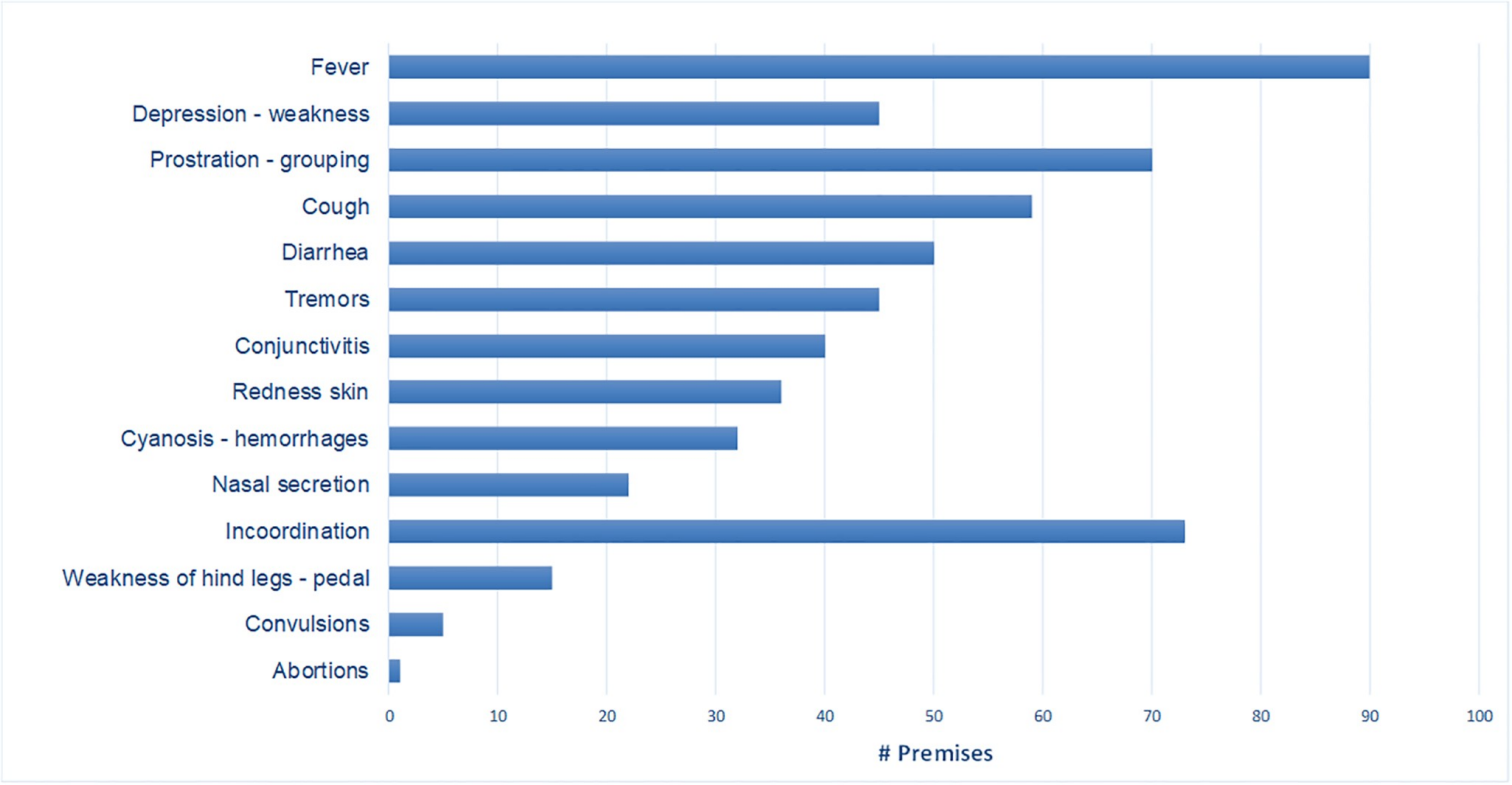
Frequency of clinical signs in the 134 outbreaks of CSF.

High fever was the most frequent clinical sign and was observed in 90 premises (67%), with incoordination of movements and prostration being observed in 73 and 70 premises (54% and 52%, respectively). Other clinical signs frequently observed were cough (59 premises, 44%), diarrhea (50, 37%), tremors (45, 34%), and depression and weakness (45, 33%) (Fig 1).

### Vaccination

Out of the 134 outbreaks, 98 (73%) had not been vaccinated against CSF, 23 (17%) had been only partially vaccinated, and the other 13 (10%) occurred in vaccinated premises. In 5 of these last premises, the vaccination was carried out 4–6 days before the presentation of the clinical signs. In one of the premises the vaccination was carried out the same day as the presentation of clinical signs, and in 2 premises it was carried out after the appearance of signs. Therefore, only 5 cases appeared in previously fully vaccinated herds. Three of the seven commercial farms that were affected had not been vaccinated and the other four farms were only partially vaccinated.

CSF vaccination was suspended in the departments of the Atlantic Coast region, Santander and Norte de Santander in June 2013 (just before the start of the first outbreak) as they were included in an eradication zone. When the outbreaks began in that year, 50,717 pigs from the Atlantic Coast located in affected municipalities or in a radius of 10 km around outbreaks were vaccinated. In September 2014, compulsory vaccination was set up again in the departments of the Atlantic Coast and in North Santander. A total of 980,752 pigs were vaccinated in the Atlantic Coast region and 53.547 in Norte de Santander in 2015, and in the consecutive years, about one and a half million animals were vaccinated yearly. In Santander, vaccination was reestablished in 2015 when the first CSF outbreaks occurred and was maintained until 2016 when it was stoped. In that period 108,775 pigs were vaccinated enabling a 90% of vaccination coverage [16].

### Spatio-temporal analysis

**Fig 2:**
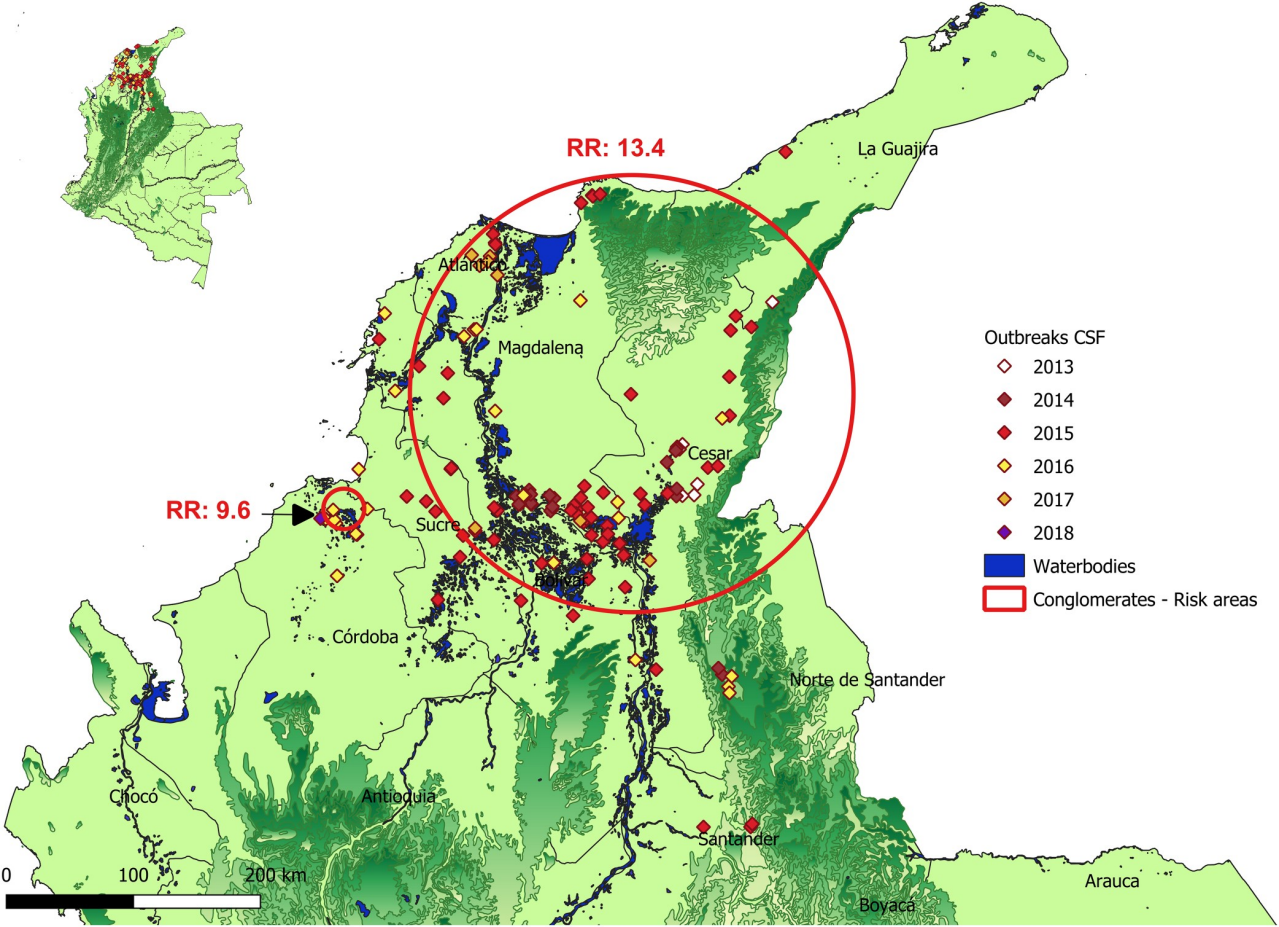
Areas of higher risk of infection with CSF.

The spatiotemporal analysis detected two statistically significant (p <0.05) clusters (or zones of greater risk). The first one with a radius of 154.5 km, included part of the departments of Atlántico, Bolívar, Cesar, La Guajira, Norte de Santander, Magdalena and Sucre, with 5.6 outbreaks more than expected, and with a relative risk (RR) of 13.4, for the period between 2014 and 2016. The second cluster had a radius of 14.2 km and grouped municipalities from the north of the department of Córdoba. The RR for the year 2016 was 9.6 due to the detection of 9.02 cases more than expected (Fig 2).

## Discussion

Six years after the appearance of the last 3 outbreaks of CSF (June 2007 in La Guajira), the disease reappeared in the area of the Atlantic Coast, producing 134 outbreaks and causing a redirect of the health strategies that had been applied until that moment.

The origin of the first outbreak in 2013 was believed to be the illegal importation of infected pigs from Venezuela. This was based on the permeability of the border and the difference in pig prices between both countries at that time due to the socio-economic situation in Venezuela. The importance of the illegal trade between both countries is unknown, but the confiscation of 48.8 tons of pork meat and 778 live smuggled pigs by the Tax and Customs Police of Colombia in Guajira, Norte de Santander, Arauca, Bogota D.C., and Cesar between 2013 and 2018 [17] suggests that it was not negligible.

Some conditions can explain the spread of the disease in these 9 departments. The most important is the high number of backyard premises. Backyard is the predominant production system in the Atlantic Coast and it is characterized by its poor management and health practices that favour spread of CSFV [18].

Much of the premises have free-range animals that leave free in the swamps until they have the right weight to be sold. Swamps occupy several departments of the Atlantic Coast and have a system of lagoons interconnected by pipes that in times of drought can be crossed by pigs. That was especially important between 2014 and 2016 due to meteorological disturbances produced by the El Niño phenomenon. This led to an increase of contacts between backyard pigs and eventually feral pigs together with an increase of animal movements on the Atlantic Coast to supply pastures. Backyard piggeries in the swamps of the Atlantic Coast and Magdalena River probably played an important role in the maintenance of CSF infection, and its dissemination to other areas, similar to the role of wild boars in Germany, transmitting either directly or indirectly CSFV to domestic pigs [6].

At the same time, pig movements between backyard premises are mostly out of control, and based on an informal pig trade, where pig traders go from municipality to municipality buying and selling pigs. Thirty-nine per cent of the outbreaks were attributed to animal movements, with this proportion being higher than the 13% of cases described in Germany in the 1990s [6], in Catalonia in 2001/02 [19] and the 17% reported in the Netherlands outbreaks [20]. This difference is probably due to the idiosyncrasy of the backyard producers that usually move their animals between neighboring properties or through local merchants without any sanitary control.

Movements of people were the supposed cause of 37% of the outbreaks, also higher than the 23% described in Catalonia [19], the 10% obtained in Germany [6] and the 6% of the outbreaks occurred in Netherlands during the high risk period, or the 15% after the implementation of measures.[20]. The main reason for this transmission was the low or no biosecurity in the backyard premises, where traders or other people entered pig pens without changing clothes, boots, or using disinfectants [1].

Other possible routes of transmission that have been described are by livestock trucks, contaminated swill feeding, movements of domestic animals other than pigs and neighboring premises, as have also been identified in commercial farms in Germany, Spain, and the Netherlands [6,19,20]. These routes were not relevant in Colombia, probably due to the particularities of the backyard piggeries. The role of the neighbors, despite being important, is not reflected, since most of them are also part of the same epidemiological unit.

Only 31 (23%) outbreaks were linked with other affected premises. The most frequent cause of virus spread was the illegal movement of infected pigs (informal trade between backyard piggeries or pigs grazed together in swamps). The second most important link with affected herds were the entry of people or trucks, which is consistent with findings of Fritzemeier et al, [6] in secondary outbreaks in Germany and with that observed in the epidemic of CSF in Netherlands [21].

One of the problems of the disease control in the region was the time elapsed between the inspection by the veterinary service and the official declaration of the outbreaks. The median time was 15 days, much higher than in other emergencies (19, 20). During this period, the premises were under quarantine but no other actions were taken, allowing the spread of the disease. The reason for this delay was the location of this laboratory in a CSF free zone. Due to protocol rules, samples were first sent to a laboratory located in Cucuta (Norte de Santander -vaccination zone) to inactivate the virus. In the last two years, a more efficient transport of samples has reduced the time to 8 days and 4 days in 2017 and 2018, respectively. Other measures, such as the involvement of more laboratories in the diagnosis, and new protocols were implemented to reduce this period.

The median time between the presentation of clinical signs and notification to the official veterinary service was 11 days, indicating that it takes several days to identify clinically or to suspect that they can be due to CSF. There may be two reasons that could influence this delay. The first one was the low number of animals in some premises, and the consequent difficulty for suspecting of an infectious disease; and the second one, the atypical or unspecific clinical signs that were present in most cases. This is compatible with Elbers et al.[22] who also identified limitations in the CSF suspicion report due to the lack of knowledge of the early signs of CSF. The owner of the animals made more than half of the suspicions. One of the explanations of this high number is that backyard producers do not have access to private veterinarians, and when they need advice, they contact the official services.

Despite that vaccination is compulsory in the area, 73% of the affected premises were not vaccinated. Veterinary authorities and the association of pork producers have made big efforts in the last 7 years, but the real vaccination coverage remains one of the main challenges in the zone. According to these organizations, a high vaccination coverage of up to 90% was achieved, but there are still an unknown number of premises that remain unnoticed, and without vaccination. The difficulties associated with vaccination coverage in remotes villages from rural areas and backyard premises has also been described by other authors [5,23]. In China, Luo et al [23], reported the incomplete vaccination coverage in remotes villages as one of the causes of CSF spread.

Twenty-seven percent of the affected premises had totally or partially vaccinated the animals. In some cases, disease occurred in weaning pigs due to the loss of colostral immunity [24]. Other reasons for failures in the vaccination process may be due to the application of the vaccine to healthy but infected animals, incomplete vaccination coverage in remote villages, or problems with the availability of the cold chain in remote areas.[5,23].

CSF affects mainly backyard piggeries, with the same proportion (95%) that was described in Bulgaria. In this country they were responsible for infecting other backyards (13% infections), but they have a low impact on the transmission to commercial pig farms [25]. The CSF Eradication Plan in the Americas recognized that family pig producers with their small number of animals per owner but with a wide dispersion of premises, make disease control difficult.[26]. Likewise, Vargas-Teran et al. [27] pointed out that the geographical distribution of CSF in the Americas is related to the backyard production system, especially the extensive open field rearing systems with minimal care and feeding.

The seven commercial farms that were infected by CSF had important failures in the vaccination. In three premises, animals had not been vaccinated against CSF, and the other four had been vaccinated only partially. The introduction of the virus was due to biosecurity failures (introduction of infected pigs, people, or domestic animals who acted as carriers). The situation in the backyard production is more complex, as despite the resources devoted to achieving a good vaccine coverage based on the application of the vaccine for free in these premises, a significant proportion of animals remain susceptible. Continuous efforts are made in the zone to update the census and to reach all premises.

Strengthening biosecurity measures in backyard farms is complex. In addition, they are mostly located in areas of waterbodies or marshes where pigs can be reared in close contact with free pigs. In this context, education campaigns on the disease ditrected to pig farmers and traders play an important role to ensure responsible pig ownership. Another important factor is the strengthening of vaccination strategies and epidemiological surveillance. Efforts should be done in high-risk areas and remote places to register populations that were previously unnoticed. The number of visits of people in charge of vaccination campaign to premises should be increased in order to maintain a high proportion of the population immunized and to monitor clinical signs. Finally, regular training of veterinary services, farm veterinarians and pig farmers in the recognition of clinical signs of CSF and differential diagnosis plays an important role to improve the efficiency of the CSF control program [18,28].

Mortality was between 31% and 39%, and the attack rate was between 38% and 48% depending on the age groups, except for sows that presented values of 17% and 21%, respectively. It can be attributable to the routine vaccination of adult animals, compared with piglets and with boars, which, in some cases are not vaccinated due to management problems. In the piglets’ case, these are not vaccinated until they are older than 55 days of age, and in fatteners, vaccination or revaccination is not carried out because they are close to going to market. Several authors have shown that the severity of the disease depends on several factors such as infection and virulence, and host factors, such as age, genetic background, nutritional condition, and immune competence. [3,29,30]; therefore, the morbidity and mortality that has been observed in the outbreaks is consistent with the presentation with an acute disease course.

The most frequent clinical signs described in the premises were fever, incoordination, prostration, grouping, cough, diarrhea, tremors, decay, weakness, conjunctivitis, and reddening of the skin, which are common with the acute form of the disease, as had also been observed in outbreaks in Spain (Catalonia), Belgium, the Netherlands, and Cuba [3,19,20,29,31]. In a small proportion of premises only respiratory, nervous, hemorrhagic, and digestive signs were observed, which are consistent with the typical signs of the disease. [3,29].

In the spatiotemporal analysis, two clusters with a higher risk of infection were identified. The first and largest included a large part of the departments of Atlántico, Bolívar, Cesar, Magdalena, and Sucre, with a relative risk (RR) of 13.4 for the years 2014 to 2016. This is consistent with the endemic peak of the disease where 115 outbreaks occurred during these 3 years, and the advance of the infection towards the center of the Atlantic Coast and the swamps, and a second cluster grouped in municipalities of Chima and Lorica of the department of Córdoba, with a RR of 9.6 for 2016. This cluster included 9 outbreaks and its origin was due to the illegal movements of infected pigs and people.

The identification of clusters of high risk of infection with CSFV helps to strengthen and redirect the health strategies of surveillance, vaccination, and biosecurity, especially in backyard piggeries of these areas, in order to reduce the number of susceptible animals that can become infected and decrease the viral circulation of CSFV in the Atlantic Coast.

In conclusion, the Colombian epidemic of CSF was mainly related with the backyard production system. The affected region has an extremely high proportion of premises based on the subsistence economy. Despite the efforts of the veterinary services, there are no registers of an unknown number of backyard. Therefore, they are not covered by the vaccination campaigns neither the surveillance network. It is important to increase efforts to record as many premises as possible and to develop education campaigns including basic biosecurity measures, especially those related with animal movements.

## Supporting information

S1 File (XLSX)

S2 File (PDF)

S3 File (DOCX)
