# Supplementary material for: Descriptive epidemiology of classical swine fever outbreaks in the period 2013-2018 in Colombia
Source: PLoS One. 2020 Jun 17;15(6):e0234490. doi: 10.1371/journal.pone.0234490 (PMC7299363; doi:10.1371/journal.pone.0234490)
Supplement: S2 File — (PDF) [file pone.0234490.s002.pdf]

INFORMACION INICIAL DE OCURRENCIA ENFERMEDAD EN UN PREDIO

No.

USO DIAGNOSTICO Y PROCESAMIENTO

1. UNIDAD LOCAL DE VIGILANCIA

ICA	OTRA

2. IDENTIFICACION Y LOCALIZACION

NOMBRE DEL PROPIETARIO ____ NOMBRE DEL PREDIO ____

DEPARTAMENTO____________________MUNICIPIO________________________VEREDA___________________________CUADRANTE__________________

LATITUD_________________________________LONGITUD_________________________________TELEFONO O FAX______________________________

3. TIPO DE EXPLOTACION

BOVINOS	AVES	PORCINOS	EQUINOS	OVINOS-CAPRINOS
LECHE ☐	COMERCIAL POSTURA ☐	COMERCIAL CRIA ☐	LABOR ☐	COMERCIAL ☐
CARNE ☐	COMERCIAL ENGORDE ☐	COMERCIAL ENGORDE ☐	DEPORTE ☐	VIVI ENDA ☐
DOBLE PRO-POSITO ☐	COMERCIAL REPRODUCCION ☐	COMERCIAL COMPLETO ☐	REMONTA ☐	FINCA ☐
	COMERCIAL POSTURA-ENGORDE ☐	VIVIENDA ☐	PASO FINO ☐	
	TRASPATIO ☐	FINCA ☐		

TROS (CUALES) ____

4. NOTIFICACION

PROPIETARIO O ADMINISTRADOR ☐

TERCEROS ☐

VIGILANCIA ACTIVA ☐

5. CRONOLOGIA

	DIA	MES	AÑO
PRIMER ENFERMO			
NOTIFICACION			
PRIMERA VISITA MED.VET.			

PREDIOS VECINOS AFECTADOS (Diligenciar una forma 3-106 Y 3-108 en cada predio)

SI ☐ NO ☐ CUANTOS____________________________

POBLACION SUSCEPTIBLE: (Fincas vecinas a predios afectados)

ESPECIE:_____________No._______ ESPECIE:___________________No.________

ESPECIE:_____________No._______

6. POBLACION, ENFERMOS Y MUERTOS DESDE EL INICIO HASTA LA FECHA DE ESTA VISITA

	ESPECIE	POBLACIÓN			ENFERMOS			MUERTOS (1)		
		VACUNA-DOS	NO VACUNADOS	TOTAL	VACUNA-DOS	NO VACUNADOS	TOTAL	VACUNA-DOS	NO VACUNADOS	TOTAL
BOVINOS	TERNEROS (AS) < 1 AÑO									
	HEMBRAS 1-2 AÑOS									
	HEMBRAS 2-3 AÑOS									
	HEMBRAS > 3 AÑOS									
	MACHOS 1-2 AÑOS									
	MACHOS 2-3 AÑOS									
	MACHOS > 3 AÑOS									
	TOTAL									
PORCINOS	LACTANTES < 2 MESES									
	MACHOS 2-6 MESES									
	HEMBRAS 2-6 MESES									
	MACHOS >6 MESES									
	HEMBRAS >6 MESES									
	TOTAL									
	OVINOS									
	CAPRINOS									
	EQUINOS < 1AÑO									
	1-2 AÑOS									
	> 2 AÑOS									
	TOTAL									

AVES										
	POLLO DE ENGORDE									
	CRIA									
	FINALIZACION									
	**TOTAL**									
	AVES DE POSTURA									
	0-6 SEMANAS									
	7-18 SEMANAS									
	> 18 SEMANAS									
	TOTAL									
	REPRODUCTORAS									

(1) LOS MUERTOS SE CONSIDERAN QUE ENFERMARON INCLUYALOS EN LOS ENFERMOS

7. SINGNOS Y SINTOMAS

____

____

____

____

____

8. LESIONES EN LA NECROPSIA

____

____

____

____

____

9. CUADRO CLINICO PRESUNTIVO

10. ULTIMA VACUNACION CONTRA LA ENFERMEDAD SOSPECHADA ANTES DEL INICIO (VERIFIQUE)

DIA __________ MES _________ AÑO ___________ NUMERO DE LOTE ___________ TIPO DE VACUNA __________ LABORATORIO______________________

11. TOMA DE MUESTRAS

SI ☐ TIPO DE MUESTRA ____

NO ☐ LUGAR Y NOMBRE LABORATORIO DE ENVIO ________________________________

ANALISIS SOLICITADO (S) ____

	DIA	MES	AñO
TOMA			
ENVIO			

12. INGRESO DE ANIMALES O POSIBLES "VEHICULOS" DE LA ENFERMEDAD EN LOS 30 DIAS ANTES DEL INICIO

TIPO INGRESO	No.	FECHA			IDENTIFICACION DEL ORIGEN (NOMBRES)			
		DIA	MES	AÑO	FINCA-FERIA-ETC.	PROPIETARIO	MUNICIPIO	DEPARTAMENTO

13. EGRESO DE ANIMALES O POSIBLES "VEHICULOS" DE LA ENFERMEDAD EN EL LAPSO COMPRENDIDO ENTRE 30 DIAS ANTES DE LA ENFERMEDAD Y EL MOMENTO DE LA VISITA

TIPO EGRESO	No.	FECHA			IDENTIFICACION DEL DESTINO (NOMBRES)			
		DIA	MES	AÑO	FINCA-FERIA-ETC.	PROPIETARIO	MUNICIPIO	DEPARTAMENTO

14. MEDIDAS SANITARIAS RECOMENDADAS

LIMPIEZA Y DESINFECCION ☐ TRATAMIENTO MEDICO ☐ SACRIFICIO DE ANIMALES ☐ VACUNACION EN PREDIOS VECINOS NO AFECTADOS ☐

VACUNACION EN EL PREDIO AFECTADO (NO INCLUYA ANIMALES ENFERMOS O SUS CONTACTOS) ☐ INMOVILIZACION DE ENFERMOS Y CONTACTOS ☐ CUARENTENA DEL PREDIO ☐

COMUNICACION A LA UNIDAD DE SANIDAD ANIMAL HACIA LA CUAL HUBO EGRESOS DEL(OS) PREDIO(S) AFECTADO (S) ☐

CUARENTENA DEL AREA ☐ OTRAS ☐ CUAL(es) ____

15. ORIGEN PROBABLE DE LA ENFERMEDAD

16. OBSERVACIONES

NOMBRE MEDICO VETERINARIO

FIRMA MEDICO VETERINARIO

DIRECCION RESPUESTA
