# Supplementary material for: Descriptive epidemiology of classical swine fever outbreaks in the period 2013-2018 in Colombia
Source: PLoS One. 2020 Jun 17;15(6):e0234490. doi: 10.1371/journal.pone.0234490 (PMC7299363; doi:10.1371/journal.pone.0234490)
Supplement: S3 File — (DOCX) [file pone.0234490.s003.docx]

**INITIAL OCCURRENCE INFORMATION**

**DISEASE IN A FARM No.**

**1. LOCAL UNIT OF SURVEILLANCE: ICA OTHER**

**2. IDENTIFICATION AND LOCALIZATION**

OWNER'S NAME ____________________________________ NAME OF THE FARM____________________________________

DEPARTMENT________________________ MUNICIPALITY_________________________ PARISH_____________________________

LATITUDE ____________________________________LONGITUDE ____________________________________

PHONE OR FAX___________________________________

**3. TYPE OF FARM**

| COMMERCIAL FARROWING |  |
| --- | --- |
| FATTENERS COMMERCIAL |  |
| COMMERCIAL FARROW-TO-FINISH |  |
| EXTENSIVE |  |
| BACKYARD |  |
| OTHER |  |

**4. NOTIFICATION 5. CRONOLOGY (DATE dd/mm/yy**

| OWNER OR ADMINISTRATOR |  |  | FIRST DISEASED ANIMAL |  |
| --- | --- | --- | --- | --- |
| OTHER |  |  | NOTIFICATION |  |
| ACTIVE SURVEILLANCE |  |  | FIRST INSPECTION |  |

**6. POPULATION, DISEASED AND DEAD ANIMALS FROM THE BEGINNING TO THE DATE OF THIS VISIT**

|  | POPULATION | | | | DISEASED | | | DEAD | | |
| --- | --- | --- | --- | --- | --- | --- | --- | --- | --- | --- |
|  | VACINATED | NON-VACCI-NATED | TOTAL | | VACINATED | NON-VACCI-NATED | TOTAL | VACINATED | NON-VACCI-NATED | TOTAL |
| PIGLETS< 2 MONTHS |  |  |  |  | |  |  |  |  |  |
| MALES 2-6 MONTHS |  |  |  |  | |  |  |  |  |  |
| FEMALES 2-6 MONTHS |  |  |  |  | |  |  |  |  |  |
| MALES >6 MONTHS |  |  |  |  | |  |  |  |  |  |
| FEMALES >6 MONTHS |  |  |  |  | |  |  |  |  |  |
| TOTAL |  |  |  |  | |  |  |  |  |  |

1. DEAD ANIMALS SHOULD BE ALSO INCLUDED IN THE DISEASED COLUMN

**7. CLINICAL SIGNS**

**8. LESIONS IN NECROPSIED ANIMALS**

**9. PRESUMPTIVE DIAGNOSTIC**

**10. LAST VACCINATION AGAINST THE DISEASE BEFORE START (CHECK)**

DAY __________ MONTH _________ YEAR ___________ LOT NUMBER ___________ TYPE OF VACCINE __________ LABORATORY______________________

**11. SAMPLING:** YES NO

TYPE OF SAMPLE ______________________________________________________________________

NAME OF THE LABORATORY WHERE THE SAMPLES ARE SENT _________________________________________________

ANALYSIS TO BE PERFORMED _________________________________________________________________

**12. ENTRY OF ANIMALS OR POSSIBLE "VEHICLES" OF THE DISEASE IN THE LAST 30 DAYS BEFORE THE ONSET**

| TYPE ENTRY |  | IDENTIFICATION OF THE ORIGIN ( NAMES) | | | |
| --- | --- | --- | --- | --- | --- |
|  | DATE | TYPE (FARM-MARKET-...) | OWNER | MUNICIPALITY | DEPARTMENT |
|  |  |  |  |  |  |
|  |  |  |  |  |  |
|  |  |  |  |  |  |

.

**13. EXIT OF ANIMALS OR POSSIBLE "VEHICLES" OF THE DISEASE BEWEEN 30 DAYS BEFORE THE ONSET**

**AND THE DATE OF THE INSPECTION**

| TYPE EXIT |  | IDENTIFICATION OF THE DESTINATION ( NAMES) | | | |
| --- | --- | --- | --- | --- | --- |
|  | DATE | TYPE (FARM-MARKET-...) | OWNER | DATE | DEPARTMENT |
|  |  |  |  |  |  |
|  |  |  |  |  |  |
|  |  |  |  |  |  |

**14. RECOMENDED HEALTH MEASURES**

| CLEAN AND DISINFECTION |  | IMMOVILIZATION OF DISEASED AND CONTACT ANIMALS |  |
| --- | --- | --- | --- |
| TREATMENT |  | QUARANTINE IN THE FARM |  |
| SACRIFICE OF THE ANIMALS |  | COMUNICATE WHERE ANIMALS HAVE BEEN MOVED TO THE ANIMAL HEALTH UNIT RESPONSIBLE OF THE AREA |  |
| VACCINATION IN NEIGHBOUR NON-AFFECTED FARMS |  | QUARANTINE IN THE AREA |  |
| VACCINATION IN THE AFFECTED FARM (DO NOT VACCINATE DISEASED ANIMALS AND THEIR CONTACTS) |  | OTHER (INDICATE) |  |

**15. POSSIBLE ORIGIN OF THE DISEASE**

**16. OTHER REMARKS**
